# Quantifying hormone receptor status in lobular breast cancer in an institutional series: the relationship between estrogen and progesterone receptor status and outcomes

**DOI:** 10.1007/s10549-023-07059-y

**Published:** 2023-07-27

**Authors:** Elle N. Clelland, Harriet T. Rothschild, Anne Patterson, Julissa Molina-Vega, Mandeep Kaur, W. Fraser Symmans, Christopher J. Schwartz, A. Jo Chien, Christopher C. Benz, Rita A. Mukhtar

**Affiliations:** 1 School of Medicine, University of California, San Francisco, San Francisco, CA USA; 2 Department of Surgery, University of California, 1825 4th Street, 3rd Floor, Box 1710, San Francisco, CA 94143 USA; 3 Department of Pathology, MD Anderson Cancer Center, Houston, TX USA; 4 Department of Pathology, University of California, San Francisco, San Francisco, CA USA; 5 Department of Medicine, University of California, San Francisco, San Francisco, USA; 6 Cancer & Developmental Therapeutics Program, Buck Institute for Research on Aging, Novato, USA

**Keywords:** Invasive lobular carcinoma, Estrogen, Progesterone, Hormone receptor positivity, Breast cancer, Immunohistochemistry, Hormone receptor

## Abstract

**Purpose:**

Recent guidelines defined a new reporting category of ER-low-positive breast cancer based on immunohistochemistry (IHC). While low positivity of either hormone receptor is uncommon in invasive lobular carcinoma (ILC), we sought to investigate whether relatively low hormone receptor positivity was associated with tumor characteristics and patient outcomes in a single institutional cohort.

**Methods:**

We searched an institutional database for cases of stage I-III ILC with available IHC reports. Based on prior published categories in ILC, ER was classified as low, medium, or high as defined by ER staining of 10–69%, 70–89%, and ≥ 90% respectively. PR low and high tumors were defined by < 20%, or ≥ 20% staining respectively. We used chi-squared tests, t-tests, and Cox proportional hazards models to evaluate associations between ER/PR categories and tumor characteristics or disease-free survival (DFS).

**Results:**

The cohort consisted of 707 ILC cases, with 11% of cases categorized as ER low, 15.1% as medium, and 73.8% as high. The majority (67.6%) were PR high. Patients with ER low/medium expression were significantly younger, and more likely to also have PR low and/or HER2 positive tumors compared to those that were ER high. In a Cox proportional hazards model adjusting for age, stage, grade, pleomorphic histology, and treatment, ER category was not prognostic for DFS, but PR negative and PR low status each had significantly worse DFS compared to PR high status (HR 3.5, 95% CI 1.8–6.7, p < 0.001; and HR 2.0, 95% CI 1.1–3.5, p = 0.015, respectively).

**Conclusion:**

These findings highlight the relevance of quantifying ER and PR within ILC.

## Introduction

Hormone receptor status in breast cancer is an important predictive marker for treatment response and outcomes. Current guidelines recommend endocrine therapy in those with ≥ 1% estrogen receptor (ER) positivity by immunohistochemistry (IHC). However, the degree of ER positivity has been thought to imply differential sensitivity to endocrine therapy [1]. This recognition has led to a recent introduction of a new reporting category for “ER-low positive” breast cancer from the American Society of Clinical Oncology and College of American Pathologists, defined as tumors having 1–10% ER expression by IHC [2].

Subsequently, investigators have evaluated the clinical implications of such ER-low positive status, with some analyses showing no difference between ER-low and ER strongly positive tumors, and others showing that ER-low positive tumors are more similar to ER-negative tumors in regard to outcomes [3–6]. However, very little data exist evaluating the spectrum of ER positivity in the setting of patients with invasive lobular carcinoma (ILC), the second most common type of breast cancer [7].

ILC is known to be a hormonally driven tumor type, with studies showing that combined estrogen and progestin hormone therapy confers an increased predisposition to ILC specifically [8]. Prior studies show high rates of strong ER positivity in most ILC tumors; indeed strictly ER-low status (1–10% positive) is very rare in ILC, with most cases being ≥ 90% ER-positive [3]. However, there remains a range of ER positivity within ILC, yet very little data evaluating heterogeneity within ILC based on level of ER expression.

In this study, we evaluated an institutional cohort of patients with stage I-III ILC to determine whether relatively low, intermediate, or high ER positivity defined clinically distinct subsets of tumors. Additionally, we evaluated the impact of progesterone receptor (PR) expression in conjunction with ER status. We hypothesized that even within highly ER-positive ILC cases, relatively lower ER and/or PR may be associated with distinct tumor features, treatment patterns, response to therapy, and clinical outcomes.

## Methods

With approval from the institutional review board (#22-37379), we abstracted clinicopathologic data from a prospectively maintained institutional database containing treatment and outcomes data for ILC patients undergoing surgery at our institution between January 1996 and September 2019.

### Population

**Fig. 1:**
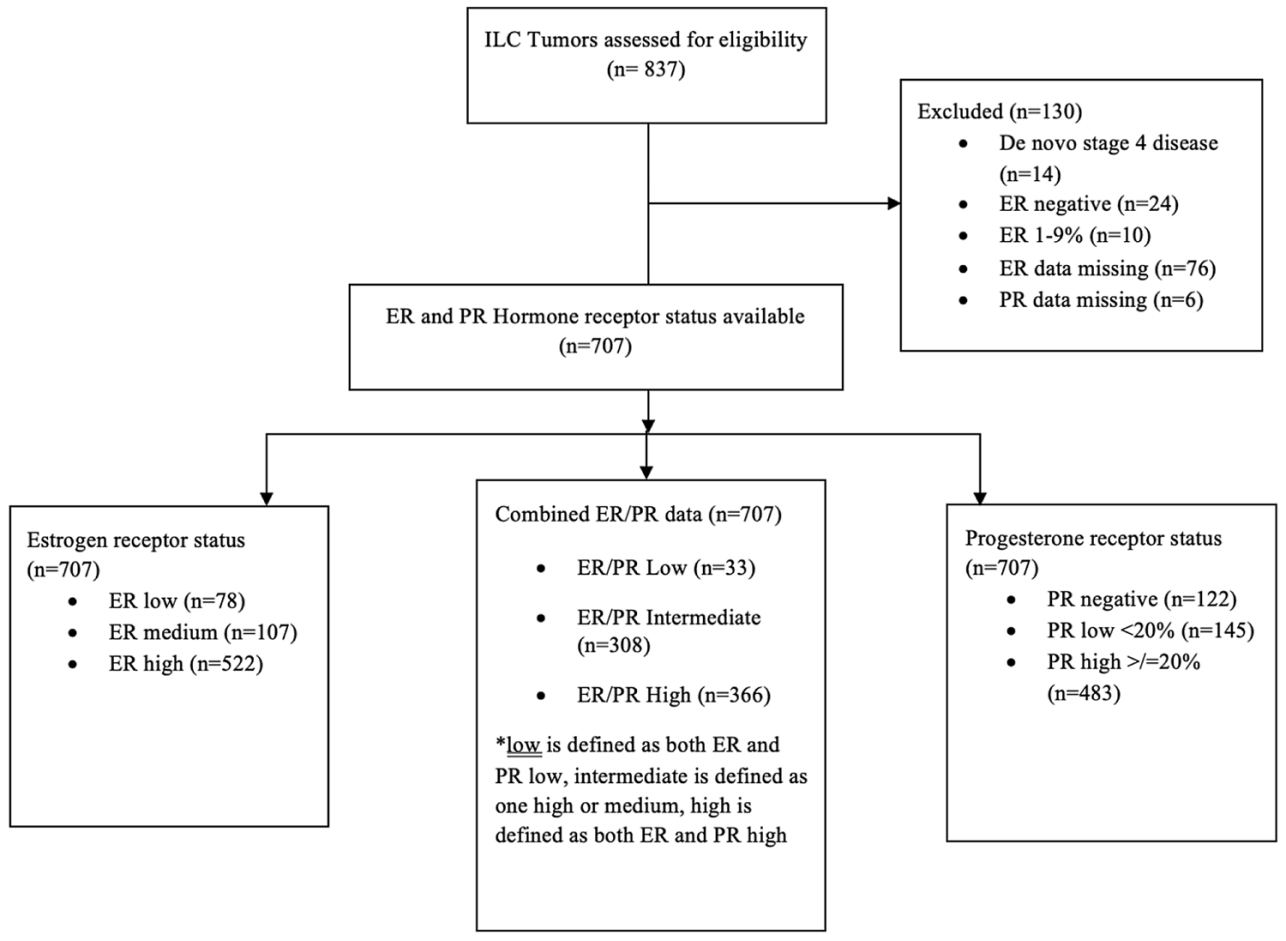
Consort flow diagram for the database population,* ER* estrogen receptor;* PR* progesterone receptor;* IDC* invasive ductal carcinoma;* ILC* invasive lobular carcinoma

We included patients with tumors that had lobular or mixed lobular/ductal histology and were diagnosed with stage I–III disease. Based on prior reported categories in ILC, we classified ER as relatively low, medium, or high expression as defined by ER staining of 10–69%, 70–89%, and ≥ 90% respectively [8]. Those with tumors ER < 10% were excluded from the analysis (Fig. 1). PR low and high tumors were defined by < 20%, or ≥ 20% staining respectively, as previously described in the literature [9]. Additionally, we evaluated combinations of ER and PR. These combined categories were classified as ER/PR low (both receptor categories low), ER/PR intermediate (one receptor category high or medium), or ER/PR high (both receptor categories high).

### Clinicopathological parameters

The following clinicopathological parameters were evaluated by ER category and PR category individually, and also in combined ER/PR categories: age at diagnosis, body mass index (BMI), tumor stage, tumor histologic grade, human epidermal growth factor receptor-2 (HER2) overexpression status, treatment (local and systemic), and recurrence outcomes. HER2 positivity was defined by 3 + staining on IHC or positive in situ hybridization and Ki-67 was defined as high if greater than 14 percent staining was present.

### Statistical analysis

We used chi-squared tests, t-tests, and Cox proportional hazards models in Stata 16.1 to evaluate associations between ER/PR categories (individually and in combination) with clinicopathologic variables, treatment, and surgical outcomes. We evaluated the association between individual ER and PR categories with disease-free survival (DFS) in multivariable models adjusting for patient age at diagnosis, tumor grade, stage, treatment, and HER2 status [10]Finally, we performed a test of interaction between ER category and receipt of adjuvant endocrine therapy to predict DFS. DFS was defined as the time from the date of diagnosis to the date of local recurrence, distant recurrence, or death; patients alive without disease recurrence were censored at the date of last follow-up*.* We used the log-rank test and Kaplan Meier method, and multivariate Cox proportional hazards models to estimate hazard ratios with 95% confidence intervals (CI) for survival analyses among those with a minimum of 6 months follow-up time with outcomes right-censored at 10 years. Data were analyzed between February 2022 and April 2022.

## Results

### Cohort characteristics

We identified 837 consecutive ILC tumors occurring in 813 patients (24 bilateral cases) between 1996 and 2019 (Fig. 1). Of these, we excluded cases with de novo metastatic disease (n = 14), those missing ER or PR status (n = 82), and those with ER positivity < 10% (n = 34), leaving 707 cases left for analysis in our study cohort (Table 1). Most tumors had classic ILC histology (n = 592), with some tumors having mixed ductal-lobular features (n = 52) or other histologic variants of ILC (n = 63). Pleomorphic histology was identified in 68 tumors. Of those pleomorphic cases, 17 were classic ILC with pleomorphic features, 1 was a mixed ILC/IDC, 1 was alveolar ILC, and the remaining 49 were categorized only as pleomorphic.

**Table 1:** Patient and tumor characteristics overall and by the combination of ER and PR; data expressed as n (%) unless otherwise specified; total n = 707

	Overall n = 707	ER/PR high n = 366	ER/PR intermediate n = 308	ER/PR Low n = 33	P value
**Age (years)**	59.6	58.8	60.3	60.5	0.23
**BMI**					
18.5–24	335 (51.6)	179 (52.0)	141 (50.9)	15 (53.6)	0.645
25–29	184 (28.4)	90 (26.2)	86 (31.1)	8 (28.6)	
≥30	130 (20.0)	75 (21.8)	50 (18.1)	5 (17.9)	
**Overall stage**					
1	436 (63.2)	223 (62.1)	192 (64.4)	21 (63.6)	0.272
2	168 (24.4)	97 (27.0)	62 (20.8)	9 (27.3)	
3	86 (12.5)	39 (10.9)	44 (14.8)	3 (9.1)	
**Tumor grade**					
1	189 (27.2)	89 (24.5)	87 (29.1)	13 (39.4)	0.045
2	473 (68.1)	262 (72.2)	194 (64.9)	17 (51.5)	
3	33 (4.8)	12 (3.3)	18 (6.0)	3 (9.1)	
**Receptor subtype**					
ER + PR + HER2-	572 (84.2)	349 (97.5)	212 (72.8)	11 (36.7)	< 0.0001
ER + PR-HER2-	76 (11.2)	0 (0)	60 (20.6)	16 (53.3)	
HER2 +	31 (4.6)	9 (2.5)	19 (6.5)	3 (10.0)	
**High Ki67 (> 14%)**	139 (38.3)	83 (40.3)	53 (37.1)	3 (33.3)	0.784
**Presence of LVI**	38 (5.6)	15 (4.2)	19 (6.4)	4 (13.3)	0.076
**Presence of LCIS**	487 (70.6)	268 (74.0)	202 (68.5)	17 (51.5)	0.014
**Type of treatment**					
Lumpectomy	120 (17.2)	50 (13.8)	66 (21.9)	4 (12.1)	0.061
Lumpectomy + rad	239 (34.2)	136 (37.5)	94 (31.1)	9 (27.3)	
Mastectomy	241 (34.5)	131 (36.1)	97 (32.1)	13 (39.4)	
Mastectomy + rad	98 (14.0)	46 (12.7)	45 (14.9)	7 (21.21)	
Neoadj chemotherapy	85 (13.5)	84 (23.3)	90 (28.7)	15 (45.5)	0.009
Adj chemotherapy	171 (24.6)	78 (21.7)	87 (28.9)	6 (18.2)	0.067

*N (%)*

*BMI* body mass index;* ER* Estrogen receptor;* PR* progesterone receptor;* LVI* lymphovascular invasion;* LCIS* lobular carcinoma in situ;* HER2* Human Epithelial Growth Factor Receptor-2;* ILC* invasive lobular carcinoma; Neg, negative; *Rad* radiation; *Neoadj* neoadjuvant;* Adj* adjuvant

Overall, the mean age at diagnosis was 59.6 years (range 21–91), and most patients had a body mass index (BMI) in the range of 18.5–25 kg/m^2^ (51.62%). There were 436 (63.2%) patients with pathologic stage I disease, 168 (24.4%) with stage II, and 86 (12.5%) with stage III disease. Most tumors were grade 2 (n = 473, 68.1%), with 189 (27.2%) being grade 1 and 33 (4.8%) being grade 3. A small proportion of cases were HER2 positive [31 of 679 with data available (4.6%)], which was consistent with prior literature [11]. The mean follow-up time was 7.4 years [standard deviation (SD) 5.9].

### Estrogen and progesterone receptor status

There were 522 (73.8%) cases with high ER (≥ 90% positive nuclei), 107 (15.1%) with medium ER staining (70–89% positive nuclei), and 78 (11.0%) with low ER staining (10–69% positive nuclei) (Table 2). Regarding progesterone receptor status, there were 478 (67.6%) with PR high (21–100% positive nuclei) and 229 (32.4%) with low PR (0–20% positive nuclei) (Table 2).

**Table 2:** Patient and tumor characteristics for ER and PR status independently. Data expressed as n (%) unless otherwise specified

	ER Low n = 78	ER Intermediate n = 107	ER High n = 522	P value	PR Low n = 229	PR High n = 478	P value
**Age (years)**	56.6	56.7	60.6	0.0006	63.6	57.6	< 0.0001
**BMI**							
18.5–24	52.4	54.3	51.0	0.107	50.0	52.4	0.763
25–29	30.2	35.1	26.8		30.2	27.5	
≥30	17.5	10.6	22.2		19.8	20.1	
**Overall stage**							
1	52 (67.5)	69 (65.1)	315 (62.1)	0.514	137 (62.3)	299 63.6)	0.018
2	20 (26.0)	23 (21.7)	125 (24.7)		45 (20.5)	123 (26.2)	
3	5 (6.5)	14 (13.2)	67 (13.2)		38 (17.3)	48 (10.2)	
**Tumor grade**							
1	32 (41.6)	31 (29.8)	126 (24.5)	0.03	59 (26.6)	130 (27.5)	0.113
2	41 (53.3)	69 (66.4)	363 (70.6)		147 (66.2)	326 (68.9)	
3	4 (5.2)	4 (3.9)	25 (4.9)		16 (7.2)	17 (3.6)	
**Receptor subtype**							
ER + PR + HER2-	47 (8.2)	77 (13.5)	448 (78.3)	< 0.001	126 (22.0)	446 (78.0)	< 0.001
ER + PR-HER2-	16 (21.1)	14 (18.4)	46 (60.5)		76 (100.0)	0 (0.0)	
HER2 +	7 (22.6)	9 (29.1)	15 (48.4)		16 (51.6)	15 (48.4)	
**High Ki67 (> 14%)**	6 (33.3)	16 (42.1)	117 (38.7)	0.818	47 (32.9)	96 (67.1)	0.615
**Presence of LVI**	6 (8.1)	8 (7.8)	24 (4.7)	0.282	17 (7.8)	21 (4.5)	0.080
**Presence of LCIS**	50 (65.8)	66 (63.5)	371 (72.8)	0.104	137 (62.6)	350 (74.3)	0.002
**Type of treatment**							
Lumpectomy	14 (17.9)	20 (18.7)	86 (16.8)	0.790	50 (22.4)	70 (14.7)	0.044
Lumpectomy + rad	21 (26.9)	35 (32.7)	183 (35.7)		69 (30.9)	170 (35.8)	
Mastectomy	30 (38.5)	35 (32.7)	176 (34.3)		69 (30.9)	172 (36.2)	
Mastectomy + rad	13 (16.7)	17 (15.9)	68 (13.3)		35 (15.7)	63 (13.3)	
Neoadj chemotherapy	19 (24.7)	18 (18.9)	48 (10.5)	0.001	36 (17.4)	49 (11.6)	0.048
Adj chemotherapy	51(65.8)	83 (77.1)	425 (81.4)	0.007	63 (28.4)	108 (22.9)	0.117

*N(%)*

*BMI* body mass index;* ER* Estrogen receptor;* PR* progesterone receptor;* LVI* lymphovascular invasion;* LCIS* lobular carcinoma in situ;* HER2* Human Epithelial Growth Factor Receptor-2;* ILC* invasive lobular carcinoma;* Neg* negative;* Rad* radiation;* Neoadj* neoadjuvant;* Adj* adjuvant

When combining ER and PR status, 366 cases (51.8%) were high for both, while 308 (43.6%) were low for either ER or PR, and 33 (4.7%) were low for both ER and PR (Table 1).

### Associations of ER and PR status with clinicopathologic factors

Patients with high ER were significantly older than those with low or medium ER (mean age 60.6, 56.7, and 56.6 years for high, low, and intermediate respectively, p = 0.0006, Table 2). In contrast, those with high PR were significantly younger than those with PR negative or low (mean age 57.6 versus 63.6 years respectively, p < 0.0001). In combination, there was no statistical significance, however those high for both ER and PR (ER/PR high) were younger than those who were ER/PR low or ER/PR intermediate (58.8 years compared to 60.5 years in ER/PR low and 60.3 years in ER/PR intermediate, p = 0.23, Table 1). ER and PR status were not associated with BMI, either alone or in combination.

ER low status was associated with both PR low status and HER2 positivity. Of the ER low cases, 42.3% were also PR low, compared to 37.4% and 29.9% of the ER medium and ER high cases respectively, (p = 0.045). HER2 was overexpressed in 10.0%, 9.0%, and 2.9% of ER low, medium, and high cases respectively (p = 0.002) (Table 2). PR low status was also associated with HER2 overexpression, with 7.3% of PR low cases also being HER2 positive, compared to 3.3% of PR high cases (p = 0.017) (Table 2).

Tumor grade was associated with ER category, but not in the expected direction. ER low tumors were significantly more likely to be grade 1 than ER medium or high tumors (41.6%, 29.8% and 24.5% grade 1 among low, intermediate, and high, respectively, p = 0.03) (Table 2). In contrast, PR status was not associated with grade in this dataset. Interestingly, when ER and PR status were combined, those with both ER low and PR low status were least likely to be grade 2, and most likely to be either grade 1 or 3 (Table 1).

While ER status was not associated with pathologic stage, those with PR low status were more likely to have stage III disease than those with PR high status (17.3% versus 10.2% stage III respectively, p = 0.018) (Table 2). Neither ER nor PR category was associated with Ki67, the presence of lymphovascular invasion, or pleomorphic subtype. However, PR low cases were significantly less likely to have associated LCIS than PR high cases (62.6% versus 74.3%, p = 0.002) (Table 2).

### Associations of ER and PR status with treatment

Overall, neoadjuvant chemotherapy was used in 85 (13.5%) cases in this study cohort, and adjuvant chemotherapy was used in 171 (24.6%) cases (Table 1). There were statistically significant differences in neoadjuvant therapy use for both ER and PR categories. For patients with ER low, medium, and high expression, neoadjuvant chemotherapy was used in 24.7%, 18.9%, and 10.5% respectively (p = 0.001) (Table 2). Among those with PR neg/low status, 36 (17.4%) received neoadjuvant chemotherapy compared to 11.6% of those with PR high status (p = 0.048) (Table 2).

Although all patients had hormone receptor positive disease, the use of adjuvant endocrine therapy was significantly lower in those with lower ER positivity. Among those with ER low tumors, only 65.8% received adjuvant endocrine therapy, compared to 77.1% of ER medium cases, and 81.4% of ER high (p = 0.007). There was no difference in the use of adjuvant endocrine therapy by PR low versus high category (Table 2).

**Table 3:** Multivariate Cox proportional hazards model for disease-free survival in invasive lobular carcinoma (ILC)

	Hazard ratio	95% CI	P
ER high (ref)			
ER low	1.01	0.50–2.02	0.98
ER medium	0.57	0.25–1.29	0.18
PR high (ref)			
PR low	2.22	1.31–3.75)	0.003
Age at diagnosis (per 1 year increase)	1.01	0.9901.04	0.25
Stage 1 (ref)			
Stage 2	0.82	0.41–1.64	0.58
Stage 3	2.43	1.24–4.74	0.009
Grade 1 (ref)			
Grade 2	0.84	0.49	0.53
Grade 3	2.55	0.91–7.19	0.076
Receipt of chemotherapy	0.96	0.52–1.75	0.89
Adjuvant endocrine therapy	0.53	0.52–1.75	0.02
HER2 Positive	1.41	0.50–3.97	0.51

Total n = 621. ER estrogen receptor; PR progesterone receptor; CIconfidence interval

The rates of mastectomy compared to breast-conserving surgery did not differ by ER or PR category; however, those with PR low status were significantly more likely to undergo lumpectomy without radiotherapy than those with PR high status (14.7% versus 22.4% respectively, p = 0.044). Interestingly, ER low status was associated with positive surgical margins at first excision (40.3% in ER low cases, 35.9% in ER medium cases, and 23.7% in ER high cases, p = 0.001). In a logistic regression model adjusting for tumor size and type of surgery, both ER low and ER medium status remained associated with significantly higher odds of positive margins compared to ER high status (odds ratio [OR] 2.4, 95% CI 1.4–3.9 and OR 1.9, 95% CI 1.2–3.0 for ER low and medium respectively). In contrast, PR status was not associated with positive margins at first excision.

### Disease free survival

**Fig. 2:**
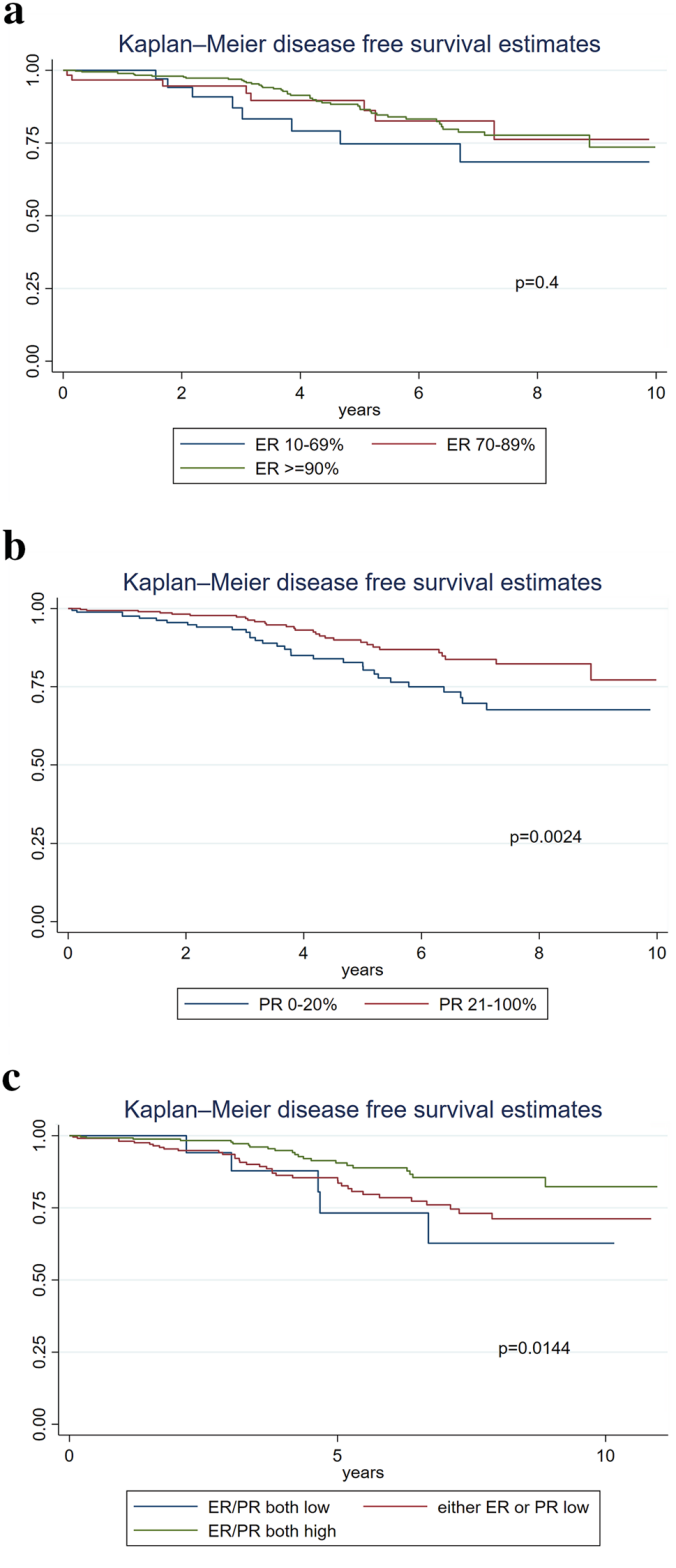
Kaplan–Meier survival curves based on receptor status (a) disease-free survival based on estrogen (ER) receptor status (b) disease-free survival based on progesterone (PR) receptor status (c) disease-free survival for combined ER/PR receptor status

There were 88 patients who experienced a recurrence event during the study period. Of those who had recurrence events, 37 (42.0%) had local recurrence, 44 (50%) had distant recurrence, 5 (5.7%) had both local and distant recurrence, and 2 (2.3%) were missing the site of recurrence. ER level was not associated with DFS, using the log-rank test (Fig. 2a). However, low PR was associated with worse DFS than high PR (p = 0.024); this was also true when excluding non-classic and pleomorphic ILC cases (p = 0.0022) (Fig. 2b). Similarly, in combination, having either intermediate ER/PR (defined as either ER or PR low) or having low ER/PR (defined as both ER and PR low) was associated with significantly worse DFS, among all cases (p = 0.014, Fig. 2c), and also when excluding non-classic and pleomorphic ILC (p = 0.0199).

In a Cox proportional hazards model for DFS adjusting for age, stage, tumor grade, receipt of chemotherapy, receipt of adjuvant endocrine therapy, and HER2 status, ER category was not associated with DFS, but PR low status remained significantly associated with worse DFS (HR 2.2, p-value 0.003, 95% CI 1.3–3.8) (Table 3). This finding remained true when the multivariable model was restricted only to patients with classic ILC histology and no pleomorphic features. When evaluating the same multivariable model using time to local recurrence and time to distant recurrence as separate endpoints, PR low status was not associated with local recurrence, but was associated with distant recurrence (HR 2.7, p-value 0.007, 95% CI 1.3–5.4).

**Table 4:** Test of interaction between ER category and adjuvant endocrine therapy showing associations with disease-free survival, adjusted for age, stage, tumor grade, PR category, HER2 status, and receipt of chemotherapy

	Hazard Ratio	95% Confidence Interval	P value
ER low without endocrine therapy (reference)			
ER low + endocrine therapy	0.14	0.04–0.48	0.002
ER medium without endocrine therapy	0.21	0.04–1.02	0.053
ER medium + endocrine therapy	0.17	0.05–0.54	0.003
ER high without endocrine therapy	0.37	0.13–1.03	0.058
ER high + endocrine therapy	0.27	0.11–0.65	0.004

This shows an association between adjuvant endocrine therapy and improved DFS across all categories of ER positivity

Of note, in a test of interaction between ER category and receipt of endocrine therapy, adjuvant endocrine therapy was associated with significantly improved DFS across ER low, medium, and high categories (Table 4).

## Discussion

In this institutional cohort of 707 cases of ILC, we found that while most tumors had ER positivity ≥ 90%, more than a quarter had lower expression of ER, as defined by prior criteria.^1^ Nearly three-quarters of cases fell into the previously described category of ER high, 15.1% into ER medium, and 11% into ER low. Compared to a large population-based analysis of patients with ILC, our study had a lower proportion of patients with ER high positive tumors. A prior study of 2512 post-menopausal patients found that 84% of ILC cases were ER high, 11% were ER medium, and 4.6% were ER low [8]. The relative shift towards more cases with lower ER positivity in our cohort likely occurred because we included both pre-and post-menopausal patients. Consistent with this idea, we found a significant association between older age and ER high positivity.

There were interesting associations between ER status and clinicopathologic variables. Those with lower ER positivity were younger, were more likely to also have low PR, were more likely to be HER2 positive, but were also more likely to have low histologic grade. ER low and medium tumors were also more likely to have positive margins at surgical excision, but ultimately the type of local therapy and DFS did not differ by ER status.

In contrast, PR status was not only associated with clinicopathologic variables but was also associated with DFS on univariate and multivariate analyses. Those with PR low status were significantly older, were more likely to be HER2 positive, more likely to have a higher stage, less likely to have associated LCIS, and had a worse prognosis.

Analysis of cases in the West German Study Group Plan B trial did not demonstrate an association between PR negativity and DFS in those with ILC [12]. In contrast, we found a significant association between lower PR expression and worse DFS; since PR negativity is uncommon in ILC, combining negative and low PR cases allows for a larger sample size for comparison. Our findings are more consistent with large database studies that demonstrate a significant association between PR negativity and worse overall survival in patients with ILC [13].

Interestingly, we did not find an association between ER/PR expression and Ki67 in the 358 patients for whom Ki67 data were available. This is consistent with data from prior studies showing a lack of correlation between ER/PR status with Ki67 in patients with ILC, but a strong inverse relationship in those with invasive ductal carcinoma [10]. Wong et al.suggest that the disruption of β-catenin signaling in ILC prevents activation of the Wnt pathway, which usually results in increased Ki67, potentially making Ki67 a less reliable predictor of behavior in ILC [10]. Of note, Ki67 was not associated with DFS in this dataset.

While recent data have identified “ER-low positive” breast cancer as a unique subset that might require different treatment approaches, these analyses have not specifically evaluated lobular tumors. Given the low prevalence of ER staining < 10% in those with ILC, there are likely very few ILC tumors represented in these studies. This is consistent with our finding that of 837 ILC cases in our institutional database, only 10 (1.2%) had ER positivity 1–9%, making the classic definition of “ER-low” less relevant for ILC. However, as other investigators have demonstrated, there is a range of ER positivity within ILC tumors. Prior studies that predominantly included patients with invasive ductal carcinoma have shown associations between ER low status and worse DFS; our findings might differ because we used a different threshold for “ER low,” versus a differential impact of ER expression on ILC than IDC. While relatively lower ER positivity was associated with different clinicopathologic features in our study, it did not seem to drive outcomes, while PR low versus high status was an important predictor of DFS in this study [14].

It is important to note the overall limitations of our study. This is a single institution dataset which may not be generalizable. Additionally, retrospective analyses are subject to treatment bias and missing information. Adherence to endocrine therapy was not available in our dataset. Furthermore, there is no clear consensus on cut points for ER low, intermediate, and high categories with several studies reporting different definitions. In this analysis, we defined ER categories based on the publication by Truin et al. [8]. Molecular subtyping was not available in our study but might be informative to better understand the relationship between Luminal A and B status, particularly with respect to PR and Ki67, Recurrence Score, and to compare with the sensitivity to endocrine therapy (SET_ER/PR_) index of endocrine-related transcriptional activity [15]. Interestingly, a large analysis found that PR status was prognostic in Luminal A but not Luminal B breast cancers [16]. Given the higher proportion of Luminal A cases among ILC, our findings may reflect this phenomenon [17]. This suggests that low PR status in ILC might identify a subset with poor prognosis despite low proliferative rates. In other work, molecular analysis of ILC cases have identified ILC specific subtypes that differ from the classic Luminal A/Luminal B framework, including the three categories of reactive-like, immune-related, and proliferative types and the two categories of immune-related and hormone-related ILC [18]^−^ [19]. A better understanding of how the spectrum of ER/PR expression by IHC fits into these ILC specific molecular subtypes would be of interest.

Overall, our findings demonstrate the heterogeneity of hormone receptor expression in ILC. While ER low and medium tumors did not have different DFS than ER high tumors, they did differ in their association with clinicopathologic variables, suggesting the need for further study into drivers of tumor development and optimal treatment. In contrast, low PR expression was an independent predictor of worse DFS.

## Conclusion

Our findings suggest there is wide clinicopathologic heterogeneity among hormone receptor status in ILC for both progesterone and estrogen. These findings broadly support the need for future research on the impact of relatively low ER and low PR in ILC to better understand these relationships and individualize treatment.

## Data Availability

The datasets generated during and/or analyzed during the current study are not publicly available due to protect patient confidentiality but are available redacted from the corresponding author on reasonable request.
